# Supplementary material for: Pipeline evaluation of a state-of-the-art AI algorithm for detection of focal cortical dysplasia: insights into potential failure sources
Source: Brain Inform. 2026 Apr 3;13(1):13. doi: 10.1186/s40708-026-00299-w (PMC13133319; doi:10.1186/s40708-026-00299-w)
Supplement: Supplementary file 3 — Supplementary Material 3 [file 40708_2026_299_MOESM3_ESM.pdf]

Supplementary Methods: Reproducible Evaluation and Correction of FreeSurfer Segmentation in the MELD Graph Pipeline

This document provides detailed operational definitions, grading criteria, rater procedures, segmentation evaluation steps, and correction workflow for FreeSurfer cortical segmentation assessment and its impact on MELD Graph predictions. It is intended to ensure full reproducibility of the failure-mode analysis described in the main manuscript.

1. Definitions and Scope

1.1 Cortical ribbon

Region between the reconstructed white matter and pial surfaces generated by FreeSurfer.

1.2 Focal FreeSurfer segmentation failure

A localized geometric inaccuracy of cortical surface reconstruction, recorded when either criterion is met:

- **Criterion A — Surface displacement:** More than 5 mm of brain tissue was excluded from the region between the true pial surface and the nearest pial surface reconstruction. Distances for the 5 mm criterion were assessed directly in Freeview [1] using the linear measurement tool on multiplanar reconstructions, explicitly avoiding oblique measurements and maintaining an orthogonal orientation relative to the true pial surface and the reconstructed pial boundary.
- **Criterion B — Lesion exclusion (patients only):** ≥50% of the cortical component of the ground-truth lesion volume outside the cortical ribbon. For feasibility, this proportion is estimated visually in Freeview by assessing the fraction of lesion voxels outside the reconstructed cortical ribbon across orthogonal planes. In most focal cortical dysplasia cases, the distinction is unequivocal (i.e., clearly above or below 50%). In uncertain cases that appear near the 50% threshold, the excluded and the total lesion portions can be delineated volumetrically by the radiologist using 3D Slicer[2], and the ratio of excluded-to-total cortical lesion volume calculated to confirm classification.

2. Segmentation Evaluation Protocol

Software: Freeview (FreeSurfer v7.2.0)[1]

Display configuration:

- T1.mgz base volume
- wm.mgz overlay (heat colormap, opacity 0.5)
- White surface (yellow)
- Pial surface (red)
- MELD cluster overlay

View (2×2 display on a 24-inch monitor):

- Axial
- Coronal
- Sagittal
- 3D surface view

3. Manual Segmentation Correction Workflow

Goal: Restore cortical ribbon segmentation at the failure site according to official FreeSurfer troubleshooting methodology[3].

Volume edited: wm.mgz

Editing principles are based on the official FreeSurfer recommendations[3]:

- Add white matter voxels where cortex excluded
- Remove white matter voxels where overexpanded
- Follow T1 anatomy
- Edit slice-by-slice in all three planes
- Verify in all planes after editing

Brush settings:

- Radius 1 voxel
- Value 255 (WM)

Editing sequential steps:

1. Load wm.mgz over brainmask/T1
2. Enable recon-edit (make sure the white matter mask is selected)
3. Identify defect extent
4. Fill the excluded white matter region
5. Erase excess white matter if needed
6. Check ribbon continuity
7. Save wm.mgz

4. Surface Reconstruction After Editing

Reconstruction stages rerun:

```
recon-all -autorecon2-wm -autorecon3
```

5. MELD Graph Prediction

Model: MELD Graph v2.2.2 [4]

Configuration: default, no harmonization

Procedure:

1. Use corrected FreeSurfer surfaces

2. Run MELD Graph prediction according to the instructions in the official documentation [4]
3. Compare clusters pre- and post-editing by analyzing the PDF reports and visually inspecting the prediction overlays in Freeview using the same settings as in the segmentation evaluation protocol

6. Reproducible FreeSurfer White Matter Editing Command Workflow

1. Freeview white matter editing:

```
freeview -v $SUBJECTS_DIR/$SUB_EDIT/mri/brainmask.mgz
$SUBJECTS_DIR/$SUB_EDIT/mri/wm.mgz:colormap=heat:opacity=0.4 -f
$SUBJECTS_DIR/$SUB_EDIT/surf/lh.white:edgecolor=yellow
$SUBJECTS_DIR/$SUB_EDIT/surf/lh.pial:edgecolor=red
$SUBJECTS_DIR/$SUB_EDIT/surf/rh.white:edgecolor=yellow
$SUBJECTS_DIR/$SUB_EDIT/surf/rh.pial:edgecolor=red
```

2. Reconstruct surfaces:

```
recon-all -autorecon2-wm -autorecon3 -subjid $SUB_EDIT
```

3. Update MELD:

```
MELD_DIR=/path/to/meld_project
cp -r $SUBJECTS_DIR/$SUB_EDIT $MELD_DIR/output/fs_outputs/
```

4. Remove old MELD derivatives:

```
rm -rf $MELD_DIR/output/fs_outputs/$SUB_EDIT/surf_meld
rm -rf $MELD_DIR/output/fs_outputs/$SUB_EDIT/xhemi
rm -f $MELD_DIR/output/fs_outputs/$SUB_EDIT/surf/*prediction*
rm -rf $MELD_DIR/output/preprocessed_surf_data/*$SUB_EDIT*
rm -rf $MELD_DIR/output/predictions/*$SUB_EDIT*
```

5. Run MELD Graph according to your installation method [4]:

References

1. Fischl B. FreeSurfer. NeuroImage. 2012 Aug;62(2):774–81. doi:10.1016/j.neuroimage.2012.01.021

2. Fedorov A, Beichel R, Kalpathy-Cramer J, Finet J, Fillion-Robin JC, Pujol S, et al. 3D Slicer as an image computing platform for the Quantitative Imaging Network. Magn Reson Imaging. 2012 Nov;30(9):1323–41. doi:10.1016/j.mri.2012.05.001

3. FreeSurfer. Making Edits to the White Matter [Internet]. 2025 [cited 2026 Feb 23]. Available from: https://surfer.nmr.mgh.harvard.edu/fswiki/FsTutorial/WhiteMatterEditsV6.0

4. Ripart, Mathilde, Spitzer H, Adler S et al. MELD Graph: MELD classifier documentation [Internet]. MELD Project; 2024 [cited 2025 Nov 18]. Available from: https://meld-graph.readthedocs.io/en/latest/index.html
